# Detection and Diagnostic Overall Accuracy Measures of Medical Tests

**DOI:** 10.5041/RMMJ.10351

**Published:** 2018-10-04

**Authors:** Gilat Grunau, Shai Linn

**Affiliations:** 1 Department of Radiology, University of British Columbia, Vancouver, British Columbia, Canada; 2 School of Public Health, Faculty of Social Welfare and Health Sciences, University of Haifa, Haifa, Israel

**Keywords:** Bayes’ theorem, clinical, diagnosis, epidemiology, medical test, overall accuracy, screening

## Abstract

**Background:**

Overall accuracy measures of medical tests are often used with unclear interpretations.

**Objectives:**

To develop methods of calculating the overall accuracy of medical tests in the patient population.

**Methods:**

Algebraic equations based on Bayes’ theorem.

**Results:**

A new approach is proposed for calculating overall accuracy in the patient population. Examples and applications using published data are presented.

**Conclusions:**

The overall accuracy is the proportion of the correct test results. We introduce a clear distinction between the overall accuracy measures of medical tests that are aimed at the detection of a disease in a screening of populations for public health purposes in the general population and the overall accuracy measures of tests aimed at determining a diagnosis in individuals in a clinical setting. We show that the overall detection accuracy measure is obtained in a specific study that explores test accuracy among persons with known diagnoses and may be useful for public health screening tests. It is different from the overall diagnostic accuracy that could be calculated in the clinical setting for the evaluation of medical tests aimed at determining the individual patients’ diagnoses. We show that the overall detection accuracy is constant and is not affected by the prevalence of the disease. In contrast, the overall diagnostic accuracy changes and is dependent on the prevalence. Moreover, it ranges according to the ratio between the sensitivity and specificity. Thus, when the sensitivity is greater than the specificity, the overall diagnostic accuracy increases with increasing prevalence, and vice versa, that is, when the sensitivity is lower than the specificity, the overall diagnostic accuracy decreases with increasing prevalence so that another test might be more useful for diagnostic procedures. Our paper suggests a new and more appropriate methodology for estimating the overall diagnostic accuracy of any medical test. This may be important for helping clinicians avoid errors.

## INTRODUCTION

The accuracy of medical tests is important for minimizing errors and their possible sequelae. “Accuracy of a diagnostic test” is a term that is frequently used loosely to describe the evaluation of a medical test versus a gold standard^1^–^16^—for example, the detection (in the general population) or diagnosis (in the patient population) of cardiovascular disease using a stress test versus catheterization as a gold standard. Similarly, such an evaluation is performed using prostate-specific antigen (PSA) for detecting or diagnosing prostate cancer versus a biopsy as a gold standard. Some textbooks and publications use a specific measure of “overall accuracy” of a test that is the ratio of correct diagnoses to all diagnoses (correct and incorrect) in a 2×2 table. It is thus the proportion of the correct test results.^1^

Alberg et al.^17^ recommended cautious use of the overall accuracy measure, because it does not take into account the true prevalence of the disease and therefore is misleading. A similar cautious approach was also advocated by others.^18^–^20^ Our manuscript addresses this problem.

We suggest that there should be a clear distinction between the overall accuracy measures of a test aimed at the *detection* of a disease in a screening setting in a population for public health purposes in the general population and the overall accuracy measures of a test aimed at determining a *diagnosis* of individuals in a clinical setting in the patient population. The overall detection accuracy measure is obtained in a specific study that samples persons with known diagnoses, and may be useful for public health screening tests. It is different from the overall diagnostic accuracy that is calculated in the clinical setting, sampling individuals with a positive or a negative test result. We thus suggest using two distinct overall accuracy measures: the *overall detection accuracy*, which is applicable in the screening and public health settings, and the *overall diagnostic accuracy*, which is applicable in the clinical setting and is dependent on the prevalence of the disease (that is, the proportion of persons with the disease). This new measure may be important for helping clinicians avoid errors.

## DETECTION MEASURES IN A SELECTED STUDY POPULATION (TABLE 1)

The assessment of a diagnostic test is frequently based on a study in a selected population, sampled according to the disease status, and is determined according to the gold standard. The study is used for calculating the sensitivity and specificity (see Table 1).^1^–^16^ Note that the sampling for Table 1 is according to disease status (sick [*S_POS_*] versus not sick [*S_NEG_*]), and thus only the totals in the columns are meaningful. The data in this table are defined by the test performance among already diagnosed persons (with or without a disease). These data are important for detecting a disease in a population and are useful in a public health setting and for decision making. For example, one may evaluate how many of the sick and healthy persons may be detected by a test for a disease among passengers in a transportation vehicle, and thus assess the resources needed in various public health and disease control settings. Such data are useful for choosing the appropriate (that is, the most efficient and least costly) test in a given population with a known and constant disease prevalence.

**Table 1:** Evaluation of the Detection of a Disease in a Specific Study, Using Disease-oriented Sampling. Lower-case Letters Describe the Study Population. The vertical line (|) denotes “given.”

	Gold Standard		
	
		*S_POS_*	*S_NEG_*
	
Clinical Test	*T_POS_*	*a* = True Positive	*b* = False Positive

	*T_NEG_*	*c* = False Negative	d = True Negative
	
	Total	*a* + *c*	*b* + *d*
$sensitivity=P(T_{POS}|S_{POS})=\frac{a}{a+c}$			
$specificity=P(T_{NEG}|S_{NEG})=\frac{d}{b+d}$			
$\text{overall detection accuracy}=\frac{a+d}{a+b+c+d}$			

where:
a, number of persons with *S_POS_ and T_POS_* b, number of persons with *S_NEG_ and T_POS_* c, number of persons with *S_POS_ and T_NEG_* d, number of persons with *S_NEG_ and T_NEG_*

### Measures in Table 1

Sensitivity is defined as *a*/(*a*+*c*), which is the probability (*P*) of the test correctly identifying as test-positive (*T_POS_*) a patient with a sickness (*S_POS_*). This is the proportion of correct positive diagnoses among all patients with the disease (Table 1). Specificity is defined as *d*/(*b*+*d*), which is the proportion of correct negative test-based diagnoses (*T_NEG_*) among all healthy individuals without the sickness (*S_NEG_*).

Note that the prevalence of the disease in Table 1 is artificially determined by the researcher, according to the number of persons with the disease (*a*+*c*) and without it (*b*+*d*) among those selected for the study, as seen in the table.

The (artificial) study prevalence in Table 1 is thus:

(Eq. 1) $$prevalence_{Table1}=\frac{a+c}{a+b+c+d}$$

Note that this is not the disease prevalence in the patient population of interest but rather that in the specific study population determined solely by the researcher. These numbers are artificially determined by the researcher in specific studies in which persons with and without a known diagnosis of a disease are sampled, and they may be influenced by a myriad of considerations, including budget, availability of patients, convenience, and time limitations. Thus, the sensitivity and specificity are not important for clinicians, as these are measured in artificial data and have no relevance to the diagnosis or treatment of patients.

### “Overall Detection Accuracy” of a Diagnostic Test Calculable in a Specific Study Population (Table 1)

The overall accuracy of a diagnostic test is commonly calculated in a specific study as:

(Eq. 2) $$\text{Overall detection accuracy}=\frac{a+d}{a+b+c+d}$$

This overall accuracy measure indicates the overall detection of persons with or without a disease in a population. It indicates how many persons with and without a disease could be correctly identified and is dependent on the disease prevalence in the specific sample used, which is artificially determined and could be different from the true prevalence of the disease in the entire study population. Thus, it is not necessarily transferrable to other populations with a different prevalence of the disease.

This measure can be written in another way (for the derivation, see the Additional Material). Let us observe the (artificial) disease prevalence odds (*x*) in the study:

(Eq. 3) $$\frac{a+c}{b+d}=x$$

i.e.:

$$\begin{array}{l} \frac{\frac{a+c}{a+b+c+d}}{\frac{b+d}{a+b+c+d}}=\frac{prevalence_{Table1}}{1-prevalence_{Table1}}\\ =\text{specific study prevalence odds ratio in Table }1. \end{array}$$

Thus,

(Eq. 4) $$\begin{array}{l} \text{Overall detection accuracy}=\frac{a+d}{a+b+c+d}\\ =\frac{sensitivity*x+specificity}{x+1} \end{array}$$

It follows that the commonly used “accuracy” or “overall accuracy” measure is in fact a weighted average of the sensitivity and specificity, with weights that are the artificially determined numbers of persons with a disease, *a*+*c*, and without a disease, *b*+*d*, who are included in a specific study.

To demonstrate this, let us consider three situations:

1. The first is a study with an equal number of persons with and without a disease, *a*+*c=b*+*d*, and thus *x*=1 (e.g. 100 sick and 100 healthy persons are studied). The (artificial) prevalence in such a study is 50%, which is rarely the true prevalence of the disease in the population of interest. In such a study, the overall detection accuracy will be in fact an average value of the specificity and sensitivity.
2. If a disease is rare, that is, if a study is designed with more persons without than with a disease, and thus *x*<1, the resulting overall accuracy measure is more heavily dependent on the specificity.
3. Conversely, for a common disease, a study designed with more persons with than without a disease, and thus *x*>1, will lead to an overall accuracy measure that is more heavily dependent on the sensitivity.

Thus, the size of the study groups leads to a biased and potentially misleading measure of the “overall accuracy” if calculated based on Table 1.

## DIAGNOSTIC MEASURES IN THE PATIENT POPULATION (TABLE 2)

The overall detection accuracy mentioned above is dependent on an artificial prevalence of the disease, as in Table 1, and thus is not applicable to an individual in a patient population. Thus, the ability of a test to diagnose a disease or the absence of a disease is evaluated in a different table that is relevant to the general patient population and the physician (Table 2). In this situation, the population is sampled according to the test results, whether positive or negative.^15^–^20^ Thus, only the total values in the rows are meaningful. As the data in Table 2 are defined by the test results (with or without pathology), they are useful in real-life clinical settings when a health professional is faced with a patient and utilizes a diagnostic test to diagnose a disease.

**Table 2:** Evaluation of Diagnostics of a Test in the General Patient Population to Which the Clinical Test Is Applied, Using Test-oriented Sampling. Upper-case Letters Describe the Patient Population. The vertical line (|) denotes “given.”

	Gold Standard		
	
		*S_POS_*	*S_NEG_*
	
Clinical Test	*T_POS_*	*A* = True Positive	*B* = False Positive

	*T_NEG_*	*C* = False Negative	*D* = True Negative
	
	Total	*A+C*	*B+D*
$\text{PPV}=\text{positive predictive value}=P(S_{POS}|T_{POS})=\frac{A}{A+B}$			
$\text{NPV}=\text{negative predictive value}=P(S_{NEG}|T_{NEG})=\frac{D}{C+D}$			
$\text{Overall diagnostic accuracy}=\frac{A+D}{A+B+C+D}$			

Where:
A, number of persons with *T_POS_ and S_POS_* B, number of persons with *T_POS_ and S_NEG_* C, number of persons with *T_NEG_ and S_POS_* D, number of persons with *T_NEG_ and S_NEG_*

### Measures in the Patient Population

The measure of interest for health providers, physicians, and patients alike is usually the positive predictive value (PPV) or negative predictive value (NPV) of the test. The vertical line (|) denotes “given” and thus *P*(*S_POS_*|*T_POS_*) denotes the probability of being sick, *S_POS_*, given that the test is positive, *T_POS_*.

The PPV is defined as:

(Eq. 5) $$PPV=P(S_{POS}|T_{POS})=\frac{A}{A+B}$$

Similarly, the NPV is defined as the success percentage when the clinical test is used to diagnose the absence of a disease:

(Eq. 6) $$NPV=P(S_{NEG}|T_{NEG})=\frac{D}{C+D}$$

Frequently, we do not have the information needed to construct Table 2 or to calculate the PPV and the NPV directly, because it is often unfeasible or unethical to perform both the diagnostic tests and an additional more invasive definitive test to determine the true diagnosis according to the gold standard (e.g. the results of a stress test would not always justify cardiac catheterization).

The translation of information on sensitivity and specificity to PPV or NPV, that is, the calculation of Table 2 from the data in Table 1, must be done using an equation based on Bayes’ theorem that uses the clinician’s prior knowledge of the probability of a disease (based on the prevalence) to calculate the probability that a test yields correct results. This equation is based on the true prevalence *P*(*S_POS_*) of the disease, that is, the probability (*P*) of the sickness (*S*) in the population (Equation 7).

(Eq. 7) PPV=P(SPOS∣TPOS)=P(TPOS∣SPOS)*P(SPOS)P(TPOS∣SPOS)*P(SPOS)+P(TPOS∣SNEG)*P(SNEG)=P(TPOS∣SPOS)*P(SPOS)P(TPOS)=sensitivity*prevalenceP(TPOS)

Note also that,

(Eq. 8) $$\text{Probability of getting a positive test}=P(T_{POS})=\frac{A+B}{A+B+C+D}$$

Similarly,

(Eq. 9) $$\begin{array}{l} NPV=P(S_{NEG}|T_{NEG})\\ =\frac{P(T_{NEG}|S_{NEG})*P(S_{NEG})}{P(T_{NEG}|S_{NEG})*P(S_{NEG})+P(T_{NEG}|S_{POS})*P(S_{POS})}\\ =\frac{P(T_{NEG}|S_{NEG})*P(S_{NEG})}{P(T_{NEG})}\\ =\frac{specificity*(1-prevalence)}{P(T_{NEG})} \end{array}$$

Let us note also that,

(Eq. 10) $$\text{Probability of getting a negative test}=P(T_{NEG})=\frac{C+D}{A+B+C+D}$$

### A Clinical Measure of Overall Accuracy, the Overall Diagnostic Accuracy Measure Calculable in the Patient Population

To estimate the average success percentage of diagnosing a disease correctly in a person in the patient population, we should calculate the overall diagnostic accuracy, which describes the accuracy of our ability to diagnose correctly a disease in the patient population, or the absence of the disease. This is calculable in Table 2 as the percentage of correct diagnoses yielded by the test:

(Eq. 11) $$\text{Overall diagnostic accuracy}=\frac{A+D}{A+B+C+D}$$

## USING SENSITIVITY AND SPECIFICITY AND THE PREVALENCE TO CALCULATE THE OVERALL DIAGNOSTIC ACCURACY

We now show that the diagnostic accuracy is based on the patient population disease prevalence together with the sensitivity and specificity. This leads to an equation that has already been developed by Alberg et al.^17^

### Application of Sensitivity in the Patient Population

The number of people with a disease who would be detected by a test in the patient population is obtained by multiplying the probability of detecting a person with a disease (the sensitivity) by the true disease prevalence in the patient population.

Thus,

(Eq. 12) Overall diagnostic accuracy of a disease in the patient population=sensitivity*prevalence

### Application of Specificity in the Patient Population

The number of people without a disease who would be detected by a test in the patient population is obtained by multiplying the probability of detecting a person without a disease (the specificity) by the true prevalence of non-disease (which is 1–*prevalence*) in the patient population.

Thus,

(Eq. 13) Overall diagnostic accuracy of no disease in the patient population=specificity*(1-prevalence)

### Overall Diagnostic Accuracy Expressed by the Sensitivity, Specificity, and the Prevalence

Thus, we can derive the overall diagnostic accuracy of the test in the patient population using the summary of the probabilities as:

(Eq. 14) Overall diagnostic accuracy=sensitivity*prevalence+specificity*(1-prevalence)

For illustration, according to this equation the overall diagnostic accuracy ranges according to the sensitivity (when the prevalence is 1) and the specificity (when the prevalence is 0). When *specificity*= *sensitivity*, the overall diagnostic accuracy is identical to both.

When the prevalence is 50%, the overall diagnostic accuracy is the average of the sensitivity and specificity.

### Inter-relationship of Prevalence, Sensitivity, and Specificity

From Equation 14, we obtain Equation 15:

(Eq. 15) $$\begin{array}{l} \text{Overall diagnostic accuracy}=sensitivity*prevalence+specificity*(1-prevalence)\\ =sensitivity*prevalence+specificity-specificity*prevalence\\ =(sensitivity-specificity)*prevalence+specificity \end{array}$$

Thus, for a test with a given sensitivity and specificity, there are three possible situations, depending on the prevalence:

- When *sensitivity*>*specificity*, the overall diagnostic accuracy **increases** with increasing prevalence
- When *sensitivity*<*specificity*, the overall diagnostic accuracy **decreases** with increasing prevalence
- When *sensitivity*=*specificity*, the overall diagnostic accuracy is constant and equals the specificity or the sensitivity, at any prevalence.

### Demonstration that Equation 14 is Identical to Equation 11

From Equation 7, we obtain Equation 16:

(Eq. 16) $$PPV*P(T_{POS})=sensitivity*prevalence$$

From Equation 9, we obtain Equation 17:

(Eq. 17) $$NPV*[1-P(T_{POS})]=specificity*(1-prevalence)$$

Thus, by combining Equation 15 and Equation 16, we obtain Alberg et al.’s equation (Eq. 18):^17^

(Eq. 18) $$\begin{array}{l} \text{Diagnostic accuracy}=sensitivity*prevalence+specificity*(1-prevalence)\\ =PPV*P(T_{POS})+NPV*P(T_{NEG})\\ =PPV*P(T_{POS})+NPV*[1-P(T_{POS})] \end{array}$$

Let us remember Equation 8 and Equation 10 above:

$$P(T_{POS})=\frac{A+B}{A+B+C+D}$$

and

$$P(T_{NEG})=\frac{C+D}{A+B+C+D}$$

Thus, substituting *P*(*T_POS_*) and *P*(*T_NEG_*) we obtain Equation 19:

(Eq. 19) $$\begin{array}{l} PPV*P(T_{POS})+NPV*P(T_{NEG})=\frac{A}{A+B}*\frac{A+B}{A+B+C+D}+\frac{D}{C+D}*\frac{C+D}{A+B+C+D}\\ =\frac{A}{A+B+C+D}+\frac{D}{A+B+C+D}\\ =\frac{A+D}{A+B+C+D} \end{array}$$

An explanation of how to estimate the difference between the two measures of overall accuracy is provided in the Additional Material.

## SUMMARY: OVERALL DETECTION ACCURACY VERSUS OVERALL DIAGNOSTIC ACCURACY

As has been explained, the overall detection accuracy of a test that is calculable using the data of a specific study (Table 1) is not applicable to the patient population, because the prevalence of the disease is artificial and dependent on the number of persons with and without a disease who are recruited to a specific study, a choice that is made by the researcher according to cost, sample availability, and practical considerations.

In contrast, the data in Table 2 are of interest to the patient (and the physician). These data serve to answer the following clinical questions. When the test is positive, what is the probability that the patient has the disease? (Answerable by the PPV, Equation 5). When the test is negative, what is the probability that the patient does not have the disease? (Answerable by the NPV, Equation 6). Regarding the test in the patient population, the clinical question is: What is the overall diagnostic accuracy? This question is answerable by our new suggested measure in Equation 11.

In contrast to the overall detection accuracy, which is based on an artificially determined prevalence in a specific study and thus may be meaningless, we suggest that calculating the *overall diagnostic accuracy* measure based on Table 2 is informative for the patient and the physician, as will be shown in the following examples (Tables 3 and 4). Note that only when the prevalence in a specific study is identical to the prevalence in the patient population, that is, *prevalence_Table1_*=*prevalence*, is the detection accuracy identical to the diagnostic accuracy (see the Additional Material).

**Table 3:** Overall Diagnostic Accuracy is dependent on the *Prevalence* and Provides Clinically Important Information.

	Prior Suspicion of Coronary Disease		
	Low	Intermediate	High
Prevalence	5%	50%	90%
*A*	30	300	540
*B*	86	45	9
*C*	20	200	360
*D*	864	455	91
Overall detection accuracy	0.711	0.711	0.711
Overall diagnostic accuracy	0.894	0.755	0.631

The original data were provided by Sackett et al.^6^^(p94)^ (in their book, Table 10) in a study of 350 patients with a prevalence of 227/350=64.86%. In that study, *a*=137, *b*=11, *c*=90, and *d*=112. Thus, the sensitivity is 60.35% and the specificity is 91.06%, and the calculated “overall detection accuracy”=71.1 and is constant, regardless of the prevalence in the three populations. However, the overall diagnostic accuracy, which changes for each population based on the prevalence (and Bayes’ rule), is more informative.

**Table 4:** Change in the Positive Predictive Value and Negative Predictive Value, as Well as the Overall Diagnostic Accuracy, Based on *Sensitivity* 0.21 and *Specificity* 0.91 for PSA>4 ng/mL, and Estimates of Prostate Cancer *Prevalence* in Different Age Groups.

	50 Years	70 Years
Prevalence of PC	40%	80%
PPV	0.6	0.9
NPV	0.6	0.2
Diagnostic Accuracy	0.63	0.35

NPV, negative predictive value; PC, prostate cancer; PPV, positive predictive value.

## EXAMPLE USING PUBLISHED (ARTIFICIAL) DATA

Let us consider a well-known example given by Sackett et al.^6^^(pp95–8)^ of the importance of prevalence for the evaluation of three different types of patient populations, I, II, and III, with a different prevalence of the disease, 5%, 50%, and 95%, respectively. The different prevalence of the disease depends on other risk factors, such as age, gender, medical history, and family history. The example compares the exercise electrocardiogram (ECG) stress test with an angiogram as the gold standard (Table 3).

Originally, the example was designed to demonstrate the importance of prevalence for determining the *PPV* and *NPV* for a diagnostic test, that is, exercise ECG used to diagnose ischemic coronary disease.

Table 3 displays the data originally given by Sackett et al. (Table 10 in their book),^6^^(p94)^ where *a*=137, *b*=11, *c*=90, and *d*=112.

Thus, the sensitivity is 60.35% and the specificity is 91.06%, and the calculated “overall detection accuracy” is 71.14%, regardless of the prevalence in the patient population.

Note that the prevalence in this particular example is 227/350=64.9%. However, this is an arbitrary and artificial prevalence, determined by researchers in a specific study, which does not reflect the real prevalence in potential patient populations I, II, or III. Had the researchers chosen to use a different prevalence in their study, the calculated accuracy would be different. Thus, the overall detection accuracy above is neither informative nor suitable for evaluating a test in a patient population having a different disease prevalence.

Using the above data, we can calculate an appropriate Table 2 for each specific patient population using their true prevalence (see the Additional Material). Table 3 demonstrates that the overall diagnostic accuracy of the test (ECG) varies and is dependent on the prevalence used. The diagnostic accuracy is appropriate for each of the potential patient populations having a different prevalence of the disease, and may be clinically useful for the physician and the patient.

## EXAMPLE USING ACTUAL PUBLISHED SCREENING DATA

Prostate cancer is common and a frequent cause of cancer death. In the United States, prostate cancer is the most commonly diagnosed visceral cancer; in 2017, there were expected to be approximately 161,000 new prostate cancer diagnoses and approximately 26,700 prostate cancer deaths.^21^ It is the most commonly diagnosed cancer in men and the seventh leading cause of male cancer deaths.

The traditional cutoff for an abnormal PSA level in major screening studies was 4.0 ng/mL. The American Cancer Society (ACS) systematically reviewed the studies in the literature that assessed the PSA test performance.^21^,^22^ In a pooled analysis, the estimated sensitivity of a PSA cutoff of 4.0 ng/mL was 21% for detecting any prostate cancer and the estimated specificity was 91%. Autopsy series in men who died from other causes have shown a 30% to 45% prevalence of prostate cancer in men in their 50s and an 80% prevalence in men in their 70s.^22^

We thus used the above estimates of the sensitivity and specificity and a prevalence estimate of 40% at age 50 or 80% at age 70 to calculate the overall diagnostic accuracy of the PSA test (at a cutoff level of 4 ng/mL). Table 4 demonstrates that the overall diagnostic accuracy of PSA declines dramatically from 63% at age 50 to 35% at age 70. It is thus a significantly less effective test for detecting prostate cancer in older patients. This decline in the overall diagnostic accuracy conforms with Equation 12, which predicts a decline in the overall diagnostic accuracy when the sensitivity (21% for PSA) is lower than the specificity (91% for PSA).

## DISCUSSION

It is important to use accurate medical tests and thus avoid errors and unnecessary suffering and expenses. As already mentioned by Alberg et al.^17^ and others,^18^,^19^ overall accuracy measures that do not take into account the true prevalence of the disease may be misleading.

Our manuscript addresses this problem and suggests a clear distinction between the *overall detection accuracy* (which does not take the prevalence into account) and the *overall diagnostic accuracy*, which does. We suggest that the overall detection accuracy is calculable in a screening setting in populations; it may be useful for public health purposes, but it is meaningless in the clinical setting. The overall diagnostic accuracy, which is calculable in the patient population based on the true prevalence, is more informative to the patient and the physician.

Our approach adds to the current literature, in that it may clarify the use and interpretation of test results and could avoid confusion that may result from ignoring the disease prevalence in measuring the test overall accuracy. Correct evaluation of the accuracy of medical tests may be important for helping clinicians avoid errors.

## Supplementary Information



## Abbreviations

ECG: electrocardiogram
Eq.: equation (when referring to a specific equation number)
NPV: negative predictive value
PC: prostate cancer
PPV: positive predictive value
PSA: prostate-specific antigen
S_NEG_: not sick
S_POS_: sick
