# Supplementary material for: Detection and Diagnostic Overall Accuracy Measures of Medical Tests
Source: Rambam Maimonides Med J. 2018 Oct 4;9(4):e0027. doi: 10.5041/RMMJ.10351 (PMC6186004; doi:10.5041/RMMJ.10351)
Supplement: Supplementary file 1 [file 864ArticleAM.pdf]

SUPPLEMENTARY MATERIAL

This appendix has been provided by the authors to give readers additional background reading

Supplement to

Grunau G, Linn S. Detection and Diagnostic Overall Accuracy Measures of Medical Tests. Rambam Maimonides Med J 2018;9 (4):e0027. doi:10.5041/RMMJ.10351

DERIVATION FROM EQUATION 2

The overall detection accuracy is:

$$(\text{Eq. }2)\ \text{Overall detection accuracy} = \frac{a+d}{a+b+c+d} = \frac{\frac{a}{a+c}(a+c)+\frac{b}{b+d}(b+d)}{a+b+c+d}$$

$$= \frac{sensitivity(a+c)+specificity(b+d)}{(a+c)+(b+d)}$$

Dividing the numerator and denominator by *b*+*d* we get:

$$= \frac{\frac{sensitivity(a+c)}{(b+d)}+\frac{specificity(b+d)}{(b+d)}}{\frac{(a+c)+(b+d)}{b+d}} = \frac{sensitivity\frac{(a+c)}{(b+d)}+specificity\frac{(b+d)}{(b+d)}}{\frac{a+c}{b+d}+\frac{b+d}{b+d}}$$

$$= \frac{\frac{sensitivity(a+c)}{(b+d)}+specificity}{\frac{a+c}{b+d}+1} = \frac{sensitivity * x + specificity}{x+1}$$

Where:

a=true positives in the study population (number of sick persons who tested positive);

b=false positives in the study population (number of not-sick persons who tested positive);

c=false negatives in the study population (number of sick persons who tested negative);

d=true negatives in the study population (number of not-sick persons who tested negative);

x=disease prevalence odds; and

lower-case letters denote “a study population.”

UNDERSTANDING PREVALENCE

Only when the proportion of sick persons, $prevalence_s$ in a specific study is identical to the prevalence in the patient population, that is, $prevalence_{Table1} = prevalence$, is the detection accuracy identical to the diagnostic accuracy.

$$\text{(Eq. 2) Overall detection accuracy} = \frac{a+d}{a+b+c+d} = \frac{sensitivity * x + specificity}{x+1}$$

$$= \frac{sensitivity * \frac{prevalence_s}{1-prevalence_s} + specificity}{\frac{prevalence_s}{1-prevalence_s} + 1}$$

$$= \frac{sensitivity * \frac{prevalence_{Table1}}{1-prevalence_{Table1}} + \frac{specificity * (1-prevalence_{Table1})}{1-prevalence_{Table1}}}{\frac{prevalence_{Table1} + 1 - prevalence_{Table1}}{1-prevalence_{Table1}}}$$

$$= sensitivity * prevalence_{Table1} + specificity * (1 - prevalence_{Table1})$$

Only if $prevalence_{Table1}$=$prevalence$ do we get Equation 11:

$$sensitivity * prevalence + specificity * (1 - prevalence) = \frac{A+D}{A+B+C+D}$$

$$= \text{overall diagnostic accuracy (Eq. 11)}$$

Where:

A=true positives in the patient population, i.e., number of persons who tested positive who were sick;

B=false positives in the patient population, i.e., number of persons who test positive and were not sick

C=false negatives in the patient population, i.e., number of persons who test negative and were sick;

D=true negatives in the patient population, i.e., number of persons who test negative who were not sick; and

upper-case letters denote “a patient population.”

CALCULATING PREVALENCE

Calculating prevalence-specific clinical data for Table 3 based on the sensitivity and specificity for 1000 patients. Note that the prevalence for each population is A+C/1000 (5%, 50%, and 90%).

Population I

	Gold Standard		Total
	S_{POS}	S_{NEG}	
Clinical Test			
T_{POS}	A = 30	B = 86	*116*
T_{NEG}	C = 20	D = 864	*884*
Total	*50*	*950*	*1000*

Prevalence=50/1000=0.05

Overall diagnostic accuracy=894/1000=0.894

Population II

	Gold Standard		Total
	S_{POS}	S_{NEG}	
Clinical Test			
T_{POS}	A = 300	B = 45	*345*
T_{NEG}	C = 200	D = 455	*655*
Total	*500*	*500*	*1000*

Prevalence=500/1000=0.5

Overall diagnostic accuracy=755/1000=0.755

Population III

	Gold Standard		Total
	S_{POS}	S_{NEG}	
Clinical Test			
T_{POS}	A = 540	B = 9	*549*
T_{NEG}	C = 360	D = 91	*451*
Total	*900*	*100*	*1000*

Prevalence=900/1000=0.9

Overall diagnostic accuracy=631/1000=0.631

Where

S_{POS}=sick;

S_{NEG}=not sick;

T_{POS}=positive test;

T_{NEG}=negative test;

ESTIMATING THE DIFFERENCE BETWEEN THE TWO MEASURES OF OVERALL ACCURACY

By applying Equation 14 to Table 1 and Table 2, we obtain equations for the difference in the magnitude of the overall detection accuracy and the overall diagnostic accuracy:

$$\text{Difference of overall accuracy measures in Table 1 versus Table 2}$$

$$= \text{overall diagnostic ability} - \text{overall detection accuracy}$$

$$= [sensitivity * prevalence + specificity * (1 - prevalence) - sensitivity * prevalence_{Table1}$$

$$+ specificity * (1 - prevalence)]$$

$$= sensitivity * (prevalence - prevalence_{Table1}) - specificity * (prevalence - prevalence_{Table1})$$

$$= (sensitivity - specificity) * (prevalence - prevalence_{Table1})$$

Thus, for a test with a given difference between the sensitivity and specificity, the difference between the two overall accuracy measures is dependent solely on the difference in the prevalence estimates in a specific study $prevalence_{Table1}$ versus the true general patient population prevalence.
